# An Improved Method for Extracting Rat Cerebrospinal Fluid with Repeatable Large-Scale Collection

**DOI:** 10.3390/vetsci12010058

**Published:** 2025-01-15

**Authors:** Limei Wang, Wei Yang, Yanhong Ran, Hui Song, Xinxin Yan, Jianmin Guo

**Affiliations:** 1 Guangzhou Bay Area Institute of Biomedicine, Guangdong Lewwin Pharmaceutical Research Institute Co., Ltd., Guangdong Provincial Key Laboratory of Drug Non-Clinical Evaluation and Research, TCM Non-Clinic Evaluation Branch of National Engineering Research Center for Modernization of Traditional Chinese Medicine, Guangdong Engineering Research Center for Innovative Drug Evaluation and Research, Guangzhou 510990, China; 2 Division of Life Science and State Key Lab of Molecular Neuroscience, Hong Kong University of Science and Technology, Hong Kong 999077, China

**Keywords:** rat, cerebrospinal fluid, largescale, repeated collection

## Abstract

The aim of this study was to explore an improved method for extracting rat cerebrospinal fluid (CSF), observing the impact on animal health under conditions of large-scale CSF collection and evaluating the feasibility of repeated collections. A total of 20 rats were anesthetized and fixed in a stereotactic frame. A 26G scalp needle, combined with a 1 mL syringe, was used to puncture the atlanto-occipital membrane and collect approximately 170 μL of CSF. CSF was collected twice within 14 days. During the study, animals were monitored daily for food intake, body weight, and hematological parameters, and at the end of the study, histopathological examination was performed. The health of the animals remained good, and repeated CSF collections were feasible. The success rate of the procedure was 100%, with blood contamination in the CSF decreasing from 70% in the first collection to 35% in the second. This technique is convenient, accurate, and suitable for widespread applications.

## Simple Summary

This study involved anesthetizing 20 rats, which were then fixed in a stereotactic frame. A 26G scalp needle, in conjunction with a 1 mL syringe, was used to puncture the atlanto-occipital membrane for cerebrospinal fluid (CSF) collection, with an approximate volume of 170 μL. CSF was collected twice within 14 days, and during this period, daily observations were made to monitor food intake, body weight, and hematological indicators. At the end of the study, a histopathological examination was conducted. The results confirmed that large-scale CSF collection had no significant effect on the health status of the rats, and repeated collections were feasible. The success rate of the procedure was 100%, and the blood contamination of CSF decreased from 70% in the first collection to 35% in the second, demonstrating that this technique is convenient, accurate, and suitable for wider application.

## 1. Introduction

Cerebrospinal fluid (CSF) is primarily produced by the choroid plexuses located in the lateral ventricles, third ventricle, fourth ventricle, and central aqueduct. The CSF produced by the choroid plexus of each ventricle flows into the medullary cistern, also known as the cerebellomedullary cistern, which is located between the cerebellum and the medulla oblongata in the posterior cranial fossa [1,2]. CSF is a clear, colorless fluid, mostly an ultrafiltrate of plasma, with some components actively secreted by the choroid plexus. As the nutritive fluid of the central nervous system, CSF provides nutrients to neurons, removes waste products, regulates the acid-base balance of the central nervous system, buffers pressure within the brain and spinal cord, and offers protection and support to both. The secretion pressure and absorption rate of CSF primarily depend on the difference between the mean arterial pressure and intracranial pressure [3]. CSF plays a significant role in neuroscience research, particularly in studies of neurological diseases, drug delivery, and intracerebral biomarkers [4]. Especially in drug evaluation research, such as pharmacokinetic studies, the drug concentration is often very low, requiring large and repeated collections of CSF. Some drug research requires specific animals, such as genetically modified animals. As a result, CSF collection techniques have become indispensable operations in rats to meet the need of drug evaluation research.

In most cases, CSF collection is performed on rats and mice, with occasional use of dogs and rabbits [5,6]. From both cost and difficulty perspectives, rats are the most commonly used and widely applicable animals. Adult SD rats weighing approximately 300g have an estimated total CSF volume of about 580 μL, with a CSF production rate of 2.2 μL/min, meaning that theoretically, around 100 μL of CSF can be collected from a rat per hour.

Currently, several methods are used in laboratories for CSF collection, such as homemade negative pressure devices for CSF aspiration [4,7], ultrasound-guided CSF collection [8], percutaneous puncture for CSF collection [9], lumbar puncture [10], and awake CSF collection after catheter implantation [5]. These methods either require the preparation of specialized, complex devices, are associated with high mortality and low success rates, or involve uncontrolled procedures during awake states with a risk of catheter contamination, making them less suitable for routine application in CSF sample collection in laboratories. One commonly used method is CSF collection from the medullary cistern under precise visualization conditions, which is relatively easy, controllable, and has a high success rate. This method is also easy for new technicians to perform, though it still requires some pre-treatment, such as needle processing [3,4,11]. Therefore, this study plans to further optimize this method by exploring the success rate of the procedure, evaluating its potential for wider use, and investigating the volume of CSF that can be collected and its impact on animal health, as well as the feasibility of repeated collections. The goal is to collect as much CSF as needed for research purposes without negatively impacting the health of the animals.

## 2. Materials and Methods

### 2.1. Experimental Materials

Surgical instruments, biological tissue adhesive, cotton swabs, cotton balls, 0.5 mLEP tubes/1.5 mL EP tubes, thermometer, micro-injection syringes, 1 mL disposable syringes, 26G scalp needles, dry heat glass bead sterilizer, and surgical microscope were used.

### 2.2. Experimental Animals

Twenty SPF-grade SD rats, with an equal number of males and females, aged 4–20 weeks, weighing 200–450 g, were purchased from Charles River Laboratories, Guangdong, China [SCXK (Yue) 2022-0063], with the animal quality certification numbers: No. 44829700000286, No. 44829700000287, No. 44829700000290. After purchase, the animals were housed in the Guangdong Lewwin Pharmaceutical Research Institute Co., Ltd., Guangzhou, China (AAALAC international full accreditation) animal facility [SYXK (Yue) 2021-0246]. The environmental technical indicators of the animal facility meet the requirements of GUIDE. Approval for this experiment was obtained from the IACUC of Guangdong Lewwin Pharmaceutical Research Institute Co., Ltd.

### 2.3. Surgical Procedure

The animals were divided into two groups based on the volume of CSFto be collected: Group A (50–105 μL) and Group B (115–165 μL). Anesthesia was induced using isoflurane inhalation, with an oxygen flow rate set at 2 L/min and anesthesia concentration at 2–3%. Induction was performed at 1.5 L/min, and maintenance was carried out at 0.5–0.9 L/min, with additional local anesthesia using 0.5% lidocaine solution. Once the animal entered the surgical anesthesia stage, it was fixed in a stereotactic frame, and the ear bars were adjusted so that the head was tilted downward, forming an angle of approximately 135° with the spine. The skin over the head and neck was disinfected, and a longitudinal incision (about 2 cm) was made at the midpoint of the line connecting the two ear roots. The skin and superficial muscles were cut open, and a retractor was placed to widen the wound. Then, the upper muscle layers of the atlanto-occipital membrane were bluntly separated along the midline, exposing the membrane. Hemostasis during the dissection was achieved using cotton swabs and hemostatic clamps.

### 2.4. CSF Collection Procedure

Aspiration Procedure: Using a microsyringe, draw the desired volume of water (e.g., 50 μL) and inject it into an EP tube. Connect a 1 mL syringe to a 26G scalp needle and aspirate the liquid from the EP tube. Once the liquid reaches the water column level within the scalp needle, mark the 50 μL scale and simultaneously mark the syringe’s position during aspiration. Empty the syringe and pipeline of water, then clamp the pipeline to prevent air entry. Aspirate the syringe to the marked position.

Injection Procedure: Orient the bevel of the scalp needle upward and insert it at an approximately 30° angle into the atlanto-occipital membrane at a location about one-third of the distance from the upper margin of the foramen magnum. Once the needle tip penetrates the membrane, release the clamp to allow the CSF to enter the scalp needle tube due to the negative pressure. If no CSF is observed, advance the needle tip by approximately 1 mm. Upon reaching the marked scale, withdraw the scalp needle and record the volume collected.

A small amount of CSF may leak out during the initial insertion; to minimize this loss, collection started only after the needle tip was fully inserted. Once collection was complete, an equal volume of sterile saline was injected into the original puncture site at the rate of 0.2–0.3 mL/min to maintain intracranial pressure. After injection, biological tissue adhesives were used to seal the puncture site. The incision was sutured, disinfected, and the animals were subcutaneously injected with 0.2 mg/kg meloxicam and intramuscularly injected with 5 mg/kg cefotaxime sodium. The animals were maintained at 37 °C until they recovered from anesthesia.

### 2.5. Quality Assessment of CSF

The collected CSF samples were visually examined for color and turbidity. After centrifugation, blood contamination and precipitation were observed. Clear, colorless CSF with a water-like appearance was considered acceptable; CSF with blood or a yellowish color indicated blood contamination.

### 2.6. Postoperative Care

For five consecutive days after surgery, animals were given subcutaneous injections of meloxicam (0.1 mg/kg) and intramuscular injections of cefotaxime sodium (5 mg/kg) once a day. The animals were observed daily. Weigh animals and weigh their food intake regularly.

### 2.7. Blood Sample Collection and CSF Repeated Collection

Blood samples were collected from the anterior vena cava of the animals before surgery and on the 14th day post-surgery. Then, an equal amount of CSF was collected again using method 2.4 on the 14th day.

### 2.8. Euthanize Animals

Euthanize animals and collect brain tissue for histopathological examination.

### 2.9. Data Statistics

Data were expressed as “mean ± standard deviation (MEAN ± SD)” and analyzed using EXCEL software. Significant differences were assessed by Student’s *t*-test. A *p*-value of <0.05 was considered statistically significant, and a *p*-value of <0.01 was considered highly significant.

## 3. Results

**Figure 1:**
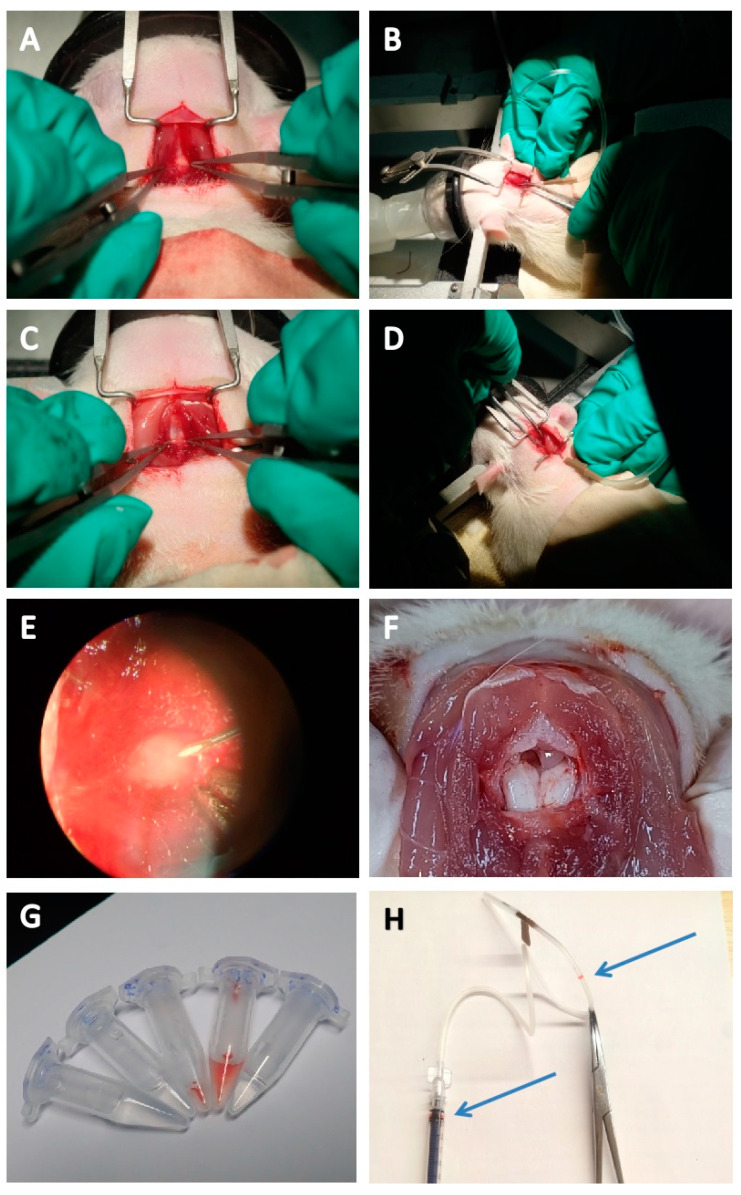
CSF collection and anatomical map of the medullary pool. (**A**): Separate the tissue and expose the occipital membrane in the first surgery; (**B**): insert needle into the occipital membrane to collect CSF in the first surgery; (**C**): separate the tissue and expose the occipital membrane in the second surgery; (**D**): insert needle into the occipital membrane to collect CSF in the second surgery; (**E**): point out the position of the occipital membrane under the microscope and the injection strategy; (**F**): dissection map of the medullary pool; (**G**): normal and blood-contaminated CSF; (**H**): syringe assembly diagram, the blue arrows show the mark during aspiration.

As shown in Figure 1A–H, the surgical procedure for separating the head and neck muscles to expose the atlanto-occipital membrane for CSF collection and the anatomical structure of the medullary pool is illustrated. The second operation is the same as the first one. Compared with the first one, due to the adhesion between the biological tissue adhesive and the muscle tissue, it is easier to bleed and cause the blurring of the window when separating the muscle, which requires timely treatment to stop bleeding and requires more detailed operation. Due to the biological tissue adhesive, the second injection into the atlanto-occipital membrane requires slightly greater force to enter.

As shown in Table 1 and Table 2, CSF was successfully collected twice from twenty SD rats, with the maximum collection volume being 175 μL. The average collection duration was 15–20 min. In the first collection, the average blood contamination rate was 70%, while the second collection had an average blood contamination rate of 35%.

As shown in Table 3 and Table 4, comparison of the blood routine indicators before and 14 days after CSF collection revealed significant differences in monocytes in female animals when the collection volume was 50–105 μL. In male animals, significant differences were observed in the total number of red blood cells and monocytes for both the 50–105 μL and 115–165 μL groups, with significant differences in neutrophils in the 115–165 μL group. White blood cells (WBC) and lymphocytes showed a slight increase 14 days after collection. No significant differences were found for other indicators.

**Figure 2:**
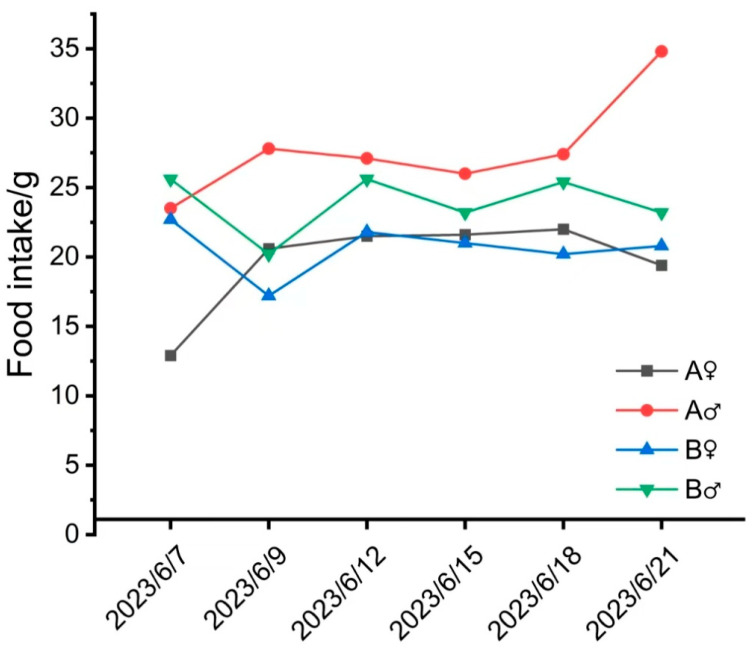
Statistics of food intake at different times after surgery.

**Figure 3:**
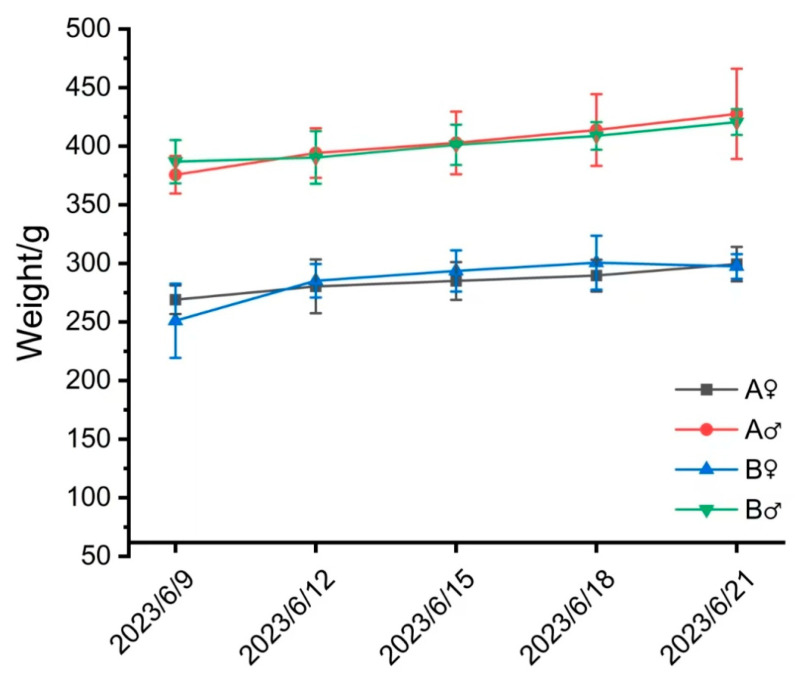
Weight statistics at different times after surgery.

Daily observation of the animals’ appearance and behavior showed no adverse reactions, and the animals were in good health. As shown in Figure 2 and Figure 3, no significant differences were observed in food intake or body weight between the different collection volume groups before and after CSF collection. All animals showed a weight-increase trend, with no abnormal weight fluctuations.

**Figure 4:**
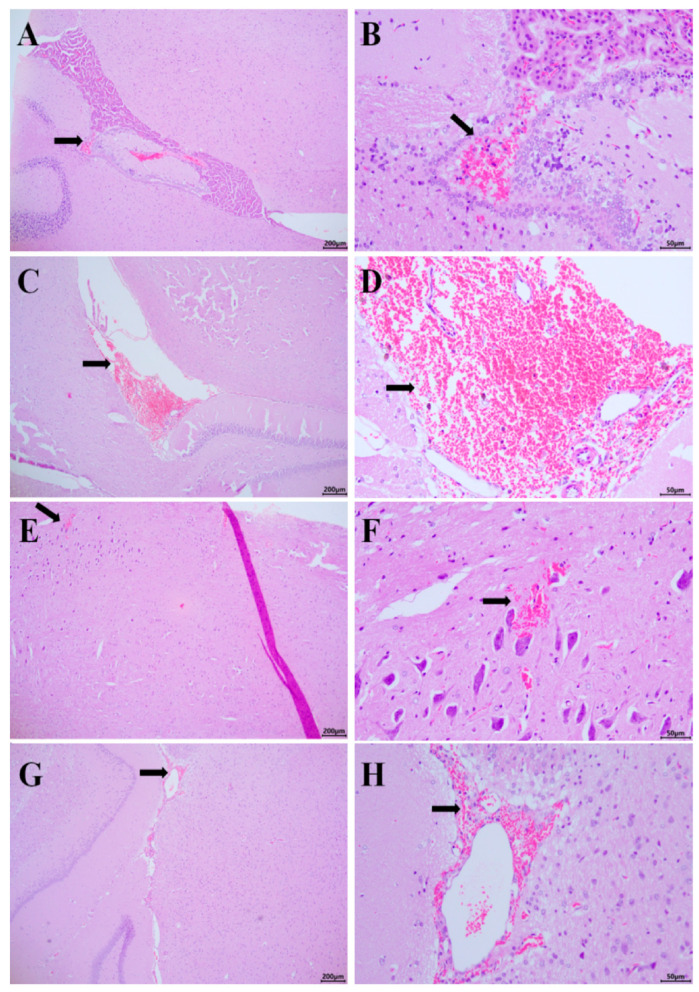
Rat brain slices (HE staining, left 40×, right 400×). (**A**,**B**): HE staining section of the fourth ventricle of A01; (**C**,**D**): HE staining section of the third ventricle of B01; (**E**,**F**): cerebellar white matter HE staining section of A09; (**G**,**H**): HE staining section of the third ventricle of B03. The arrow indicates an abnormal distribution of red blood cells.

As shown in Figure 4A–H, histopathological examination of brain tissue sections stained with HE revealed minor red blood cell infiltration around the choroid plexus of the fourth ventricle (A01), significant red blood cell infiltration in the third ventricle (B01), minimal red blood cell abnormal distribution in the cerebellar white matter (A09), and minor red blood cell infiltration in the third ventricle (B03). The remaining animals had normal meningeal structures, no vascular dilation in the meninges.

## 4. Discussion

In the CSF collection process, the following considerations must be noted: Since multiple collections are required, care must be taken to avoid damaging the skin and muscle tissues during the surgical procedure. A scalpel should be used to carefully cut through the skin and superficial muscles, followed by blunt dissection to separate the muscle layers overlying the occipital membrane. The rat’s head should be stabilized using a stereotaxic apparatus, and the body elevated with a sterile towel to align the head at a 135° angle relative to the spine, which will help expand the occipital membrane area. The superior and inferior edges of the occipital membrane should be thoroughly exposed prior to needle insertion. The central depression at the superior edge can be located with forceps, and a needle should be inserted at a 30° angle along the line connecting the depression and the inferior edge. If resistance is encountered during the collection, the needle angle should be adjusted gently, or the needle tip should be moved slightly forward or backward, ensuring that it does not exceed 0.5 mm. During collection, it is important to withdraw the syringe slowly. Rapid aspiration could result in a sudden drop in intracranial pressure, potentially causing abnormal red blood cell exudation in the subarachnoid space and ventricles. Blood contamination typically occurs during the initiation and completion of the needle insertion. To avoid introducing red blood cells during insertion, the wound area should be cleaned with a cotton swab soaked in saline followed by a dry cotton swab. After collection, a hemostat should be used to clamp the catheter at the scalp needle tip to prevent aspiration of CSF, which could lead to blood or protein contamination.

During the initial procedure, female animals were operated on first. Due to issues with maintaining stable control and incomplete insertion of the needle into the medullary cistern on the inclined side, contamination with exogenous blood occurred. Subsequent improvements focused on stabilizing the procedure, promptly removing blood stains, and ensuring the needle was fully inserted into the medullary cistern before collecting CSF, which effectively reduced the contamination rate in male animals. In comparison to the first procedure, the second operation was more prone to bleeding due to varying degrees of muscle tissue adhesion, which often led to the blurring of the observation window. This required immediate intervention to control the bleeding. Furthermore, the exposed area of the observation window was smaller, and the procedural requirements were more meticulous. As a result, the contamination rate was higher in the male animals. This prompted further refinement of the technique to ensure prompt removal of blood and stabilization of the operation, along with precise needle positioning to minimize contamination during subsequent female animals procedures.

After CSF extraction, the animals must be replenished to maintain the intracranial pressure. The supplementary fluids include sterile saline and artificial CSF. Miyajima M. et al. [12] noted that the use of artificial CSF as a perfusion fluid during third ventriculostomy was more effective than sterile saline in reducing severe host reactions. Artificial CSF was found to be more effective in maintaining cerebral homeostasis compared to sterile saline. Similarly, Mori K. et al. [13] pointed out that saline brain lavage can induce adverse neurotoxic effects, such as nerve cell death, seizures, high fever, and headache in both experimental animals and humans. Furthermore, saline irrigation has been shown to exacerbate cerebral edema around damaged brain tissue, whereas the use of artificial CSF has a less pronounced effect in this regard. Therefore, flushing and infusing the brain with artificial CSF, which has a similar electrolyte and solute composition to human CSF, appears to be a more rational choice. However, some studies have used sterile saline. For example, Cummings K.J. et al. [14] employed saline as a control group for cisterna magna injection. In their study on a rat neuropathic pain model, Leiphart J.W. et al. [15] observed that intrathecal infusion of saline had a mild analgesic effect, whereas artificial CSF did not exhibit such an effect in this context.

In this study, we injected saline at a rate of 0.2–0.3 mL/min, whereas Valitsky M. et al. [16] used an injection rate of 5 µL/h for CSF replacement therapy in mice. Bothwell S.W. et al. [17] highlighted that reducing CSF pressure can stimulate CSF secretion, although the effects of pressure gradient reduction on CSF secretion remain unclear. Changes in osmolarity can alter water flux across the choroid plexus, with ions being transported from the circulating blood into the CSF via their respective transporters, while water likely moves through a transcellular process that facilitates uphill water transport. Yamada S. et al. [18] investigated CSF flow velocity, reporting rates ranging from 0 to 9 mL/min, noting that both respiratory and heart rates affect CSF flow.

In this study, the amount of CSF collected did not exceed 30% of the total CSF volume in rats. Based on subsequent health indicators, the injection of sterile saline at a rate of 0.2–0.3 mL/min did not appear to impact the health or behavior of the animals. This is likely because the amount of sterile saline injected was relatively low, and the injection volume and rate were insufficient to cause significant effects. However, the exact amount of saline that would impact animal health, as well as the rate at which it could have an effect, requires further investigation. Many studies do not inject any substance after CSF collection, possibly due to the small volume collected. The impact of reduced CSF pressure on the animals remains an area for further study.

Post-collection handling of CSF: The CSF should be aliquoted into polypropylene tubes, with each tube filled to at least 75% of its volume. Studies have shown that if the volume is less than 50%, it could reduce the Aβ42 concentration, affecting subsequent testing results. The quality of the CSF should be evaluated within 2–4 h after collection [19]. Centrifugation conditions should be adjusted depending on the specific need. If cells need to be preserved, the sample should be centrifuged at 400× *g* for 10 min; if not, a centrifugation at 2000× *g* for 10 min at room temperature is appropriate. After centrifugation, the supernatant should be divided into aliquots and stored at −80 °C. To prevent protein degradation, repeated freeze–thaw cycles should not exceed three times. The storage temperature varies depending on the intended analysis: −15 °C to −30 °C for chemical or immunological tests, room temperature for microbiological analysis, and 2–8 °C for cellular or cytological evaluations [20]. In total, 170 μL of CSF can be collected in a single dose, and no significant abnormalities were observed in blood routine indicators between different collection volumes. Therefore, a larger amount of CSF can be collected as needed for multi-item testing.

Health Impacts on Animals: Repeated collection of CSF from SD rats every 14 days did not result in animal mortality. Observations 14 days post-collection showed that the animals’ health remained good, with normal body weight and food intake. Blood routine data revealed varying degrees of white blood cell elevation. If changes in inflammatory factors in CSF need to be measured, measures should be taken to exclude inflammatory cell changes caused by surgery. Notable differences in monocyte, red blood cell, and neutrophil counts and slight increases in white blood cells and lymphocyteswere observed, which may reflect post-surgical infection or stress responses that trigger the immune mechanism of the animal body. But the overall impact on health was minimal. Therefore, a strict aseptic technique is crucial during surgery to avoid complications.

Pathological Analysis of Brain and Cerebellum: Pathological analysis of the brain and cerebellum following CSF collection revealed red blood cell infiltration around the fourth ventricle and cerebellar white matter, likely resulting from significant granulation tissue growth at the surgical site during the second collection, along with incomplete wound cleaning. Red blood cell exudation into the third ventricle may have been caused by excessive aspiration, leading to an increased intracranial pressure and subsequent leakage of red blood cells from the choroid plexus. The results showed that only four out of the twenty rats exhibited erythrocyte accumulation, while the remaining animals showed no such accumulation. The structures of the four affected animals were otherwise normal, except for the distribution of erythrocytes. During the second operation, bleeding was more likely due to the adhesion of biological tissue glue to the muscle tissue, making muscle separation prone to bleeding. As a result, the risk of blood infiltration during the surgical procedure was higher.Thus, it is important to control the aspiration pressure during CSF collection and to implement effective hemostasis measures during repeated collections to minimize blood contamination.

Training and operator skill: The success rate for CSF collection can be improved with training and proper technique. Under ideal conditions with visualization, CSF collection from the medullary pool can achieve a success rate exceeding 90%, with an average collection time of approximately 10 min per animal. However, operator skill plays a crucial role, and inexperienced operators require training to achieve similar results. As evidenced by studies from Shamir S et al., who successfully performed CSF collection in dogs using specific anatomical landmarks, operator experience significantly impacts outcomes [6]. In our study, new operators who received prior training through literature review and hands-on practice achieved a 100% success rate after approximately 20 rats, with blood contamination reduced from 70% to 35%. This suggests that CSF collection from the medullary pool is a viable technique for novice operators, provided they receive adequate training and practice.

Innovations in collection technique: One notable innovation in this study is the use of a 26G scalp needle, which is finer and easier to insert through the occipital membrane without causing damage. Other studies have used intravenous or injection needles with modifications, but these alterations may compromise the sharpness and lead to burrs, potentially causing further damage to the animal. In this study, no modifications were necessary, and the standard insertion technique allowed for accurate and successful CSF collection with a higher success rate. Negative pressure was carefully regulated by estimating the collection volume to control the sampling process, thereby preventing excessive CSF extraction and avoiding complications such as intracranial hemorrhage caused by uncontrolled negative pressure.

Comparison with other studies: Other studies have collected larger volumes of CSF. Barthel L. et al. [3] reported an average collection volume of 207 µL in a single attempt, while Pegg C.C. et al. [11] used a semi-transparent “window” in the exposed dura mater, which allowed for the visualization of blood vessels and ensured accurate collection of 50–150 µL without neurological damage. Huang Y.L. et al. [21] performed multiple collections, each yielding 5–10 µL, with a total volume of 50 µL. Mahat M.Y.A. et al. [9] conducted pharmacokinetic testing with 50 µL collected every week for four weeks, observing no abnormalities in weight, feeding, or neurological function. In our study, we focused on collecting large amounts of CSF while monitoring animal health through body weight, food intake, daily behavior, hematological markers, and tissue pathology. This confirmed that repeated large-volume CSF collection had a minimal impact on animal health.

## 5. Conclusions

This study summarizes the progress made in ensuring the long-term survival of rats through the repeated collection of large volumes of CSF. This approach minimizes the surgical risk and has minimal impact on the animals. While a certain volume of CSF collection does not negatively affect animal health, repeated surgical procedures increase the risk of infection and complicate wound healing. Therefore, the number of surgeries should be controlled. The optimal number of procedures, the amount collected each time, and the intervals between collections need further clarification. Alternatively, more optimized single-surgery methods may be explored to meet the demand for repeated sampling.

## Figures and Tables

**Table 1:** Statistics of the first collection of CSF.

Group	Gender	Sampling Volume (μL)	Sampling Success Rate (%)	Blood Contamination Rate (%)
A	Female	37.6 ± 37.7	60%	100%
	Male	75.2 ± 19.6	80%	20%
B	Female	120.6 ± 7.0	100%	100%
	Male	150.8 ± 18.3	100%	60%

**Table 2:** Statistics of the second collection of CSF.

Group	Gender	Sampling Volume (μL)	Sampling Success Rate (%)	Blood Contamination Rate (%)
A	Female	51.6 ± 15.3	100%	40%
	Male	61.8 ± 32.6	100%	80%
B	Female	121.8 ± 30.3	100%	0%
	Male	125.4 ± 42.4	100%	20%

**Table 3:** Statistics blood routine indicators 1.

Gender	Group	Sampling Time	WBC (10^9^/L)	RBC (10^12^/L)	NEUT (10^9^/L)	LYMPH (10^9^/L)	MONO (10^9^/L)	EO (10^9^/L)	BASO (10^9^/L)
Female	A	0 d	8.67 ± 1.22	7.39 ± 0.36	0.66 ± 0.20	6.20 ± 3.40	0.51 ± 0.11	0.09 ± 0.04	0.02 ± 0.01
		14 d	9.22 ± 2.20	7.54 ± 0.20	1.04 ± 1.02	7.53 ± 3.09	0.52 ± 0.15 *	0.10 ± 0.03	0.02 ± 0.01
Female	B	0 d	9.22 ± 1.27	7.54 ± 0.31	1.04 ± 0.26	7.53 ± 1.10	0.52 ± 0.12	0.10 ± 0.03	0.02 ± 0.01
		14 d	11.43 ± 3.20	7.74 ± 0.37	0.80 ± 0.14	9.89 ± 3.14	0.61 ± 0.14	0.11 ± 0.06	0.02 ± 0.01

The asterisk “*” indicates a significant difference, * *p* < 0.05.

**Table 4:** Statistics blood routine indicators 2.

Gender	Group	Sampling Time	WBC (10^9^/L)	RBC (10^12^/L)	NEUT (10^9^/L)	LYMPH (10^9^/L)	MONO (10^9^/L)	EO (10^9^/L)	BASO (10^9^/L)
Male	A	0 d	10.52 ± 1.44	7.96 ± 0.14	1.72 ± 0.73	7.96 ± 1.13	0.70 ± 0.06	0.12 ± 0.04	0.02 ± 0.01
		14 d	12.15 ± 2.11	8.42 ± 0.26 *	1.99 ± 0.25	9.19 ± 1.83	0.83 ± 0.22 *	0.13 ± 0.05	0.02 ± 0.00
Male	B	0 d	10.52 ± 1.44	7.96 ± 0.14	1.72 ± 0.73	7.96 ± 1.13	0.70 ± 0.06	0.12 ± 0.04	0.02 ± 0.01
		14 d	12.18 ± 1.67	8.40 ± 0.26 *	2.17 ± 0.84 *	8.96 ± 1.86	0.90 ± 0.13 *	0.14 ± 0.04	0.03 ± 0.00

The asterisk “*” indicates a significant difference, * *p* < 0.05.

## Data Availability

The data presented in this study are available within the article’s figuresand table. No new data were created or analyzed in this study.
